
JOURNAL INFORMATION
==============================
NLM Title Abbreviation: Comput Sci Eng
Journal ID: Comput Sci Eng
NLM Journal ID: 100911167

Computing in Science & Engineering

ISSN: 1521-9615
EISSN: 1558-366X

ARTICLE INFORMATION
==============================
PMCID: PMC7108763
PMID: 32256222
DOI: 10.1109/MCISE.2004.1267606
Article ID: 006402CSX
Article ID: 1.1695344
Article version: 1
Subjects: Departments, Your Homework Assignment

More Models of Infection: It's Epidemic

O'Leary Dianne P.
Institute for Advanced Computer Studies and the Applied Mathematics Program, University of Maryland, College Park, MD
Print publication date: 2004 Mar
Volume: 6
Issue: 2
First page: 50
Last page: 56

Copyright year: 2004
Copyright holder: IEEE Computer Society
License: All article content, except where otherwise noted, is licensed under a Creative Commons Attribution (CC BY) license ( http://creativecommons.org/licenses/by/4.0/).
License URL: https://creativecommons.org/licenses/by/4.0/

Page count: 7

==============================
Editor: Dianne P. O'Leary, oleary@cs.umd.edu

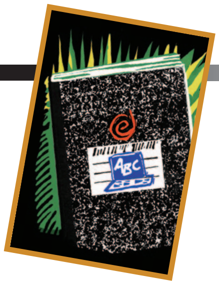

### MORE MODELS OF INFECTION: IT'S EPIDEMIC

By Dianne P. O'Leary

In the last issue, we used Monte Carlo simulations and Markov models to gain insight into a simple model of an infection's spread. We discuss this further in the solution section of this column, but first we develop some alternate models of epidemics, based on differential equations.

IN LAST ISSUE'S PROBLEM, WE STUDIED A MODEL OF AN INFECTION'S SPREAD THROUGH A HOSPITAL WARD. THE WARD WAS SMALL ENOUGH THAT WE COULD TRACK EACH PATIENT individually, but when population size grows, this kind of model becomes impractical; accordingly, we turn our attention in this issue to models that study the population as a whole.

As before, we divide the population into three groups: at day $t$, $I(t)$ is the infected proportion of the population, whereas $S(t)$ is the proportion that has never been infected. These quantities satisfy $0 \le I(t) \le 1$ and $0 \le S(t) \le 1$ for $t \ge 0$. We derive the third part, $R(t)$—the proportion of the population that was once infected but has now recovered—from the first two: $R(t) = 1 - I(t) - S(t)$.

### Models without Spatial Variation

In the models we studied before, an individual's probability of becoming infected depended on the status of his or her neighbors. In our next model, we ignore that dependence, which is equivalent to assuming a "well mixed" model: all members of the population have mutual contact.

How might we model the three groups in this population? If the infection (or at least its contagious phase) lasts $k$ days, we might assume that the recovery rate is equal to the number infected divided by $k$. Thus, on average, $1/k$ of the infected individuals will recover each day.

Let $\tau$ be the proportion of encounters between an infected individual and a susceptible one that transmit the infection. The rate of new infections should increase as any of the parameters $I$, $S$, or $\tau$ increases, so we can model this rate as $\tau I(t)S(t)$.

Next, we take the limit as the "time step" $\Delta t$ goes to zero, obtaining a system of *ordinary differential equations* (ODEs). This gives us a simple, but interesting, Model 1:

$$\frac{dI(t)}{dt} = \tau I(t)S(t) - I(t)k,$$

$$\frac{dS(t)}{dt} = -\tau I(t)S(t),$$

$$\frac{dR(t)}{dt} = I(t)/k. \qquad (1)$$

We start the model by assuming some proportion of infected individuals—for example, $I(0) = 0.005$, $S(0) = 1 - I(0)$, and $R(0) = 0$.

**Problem 1.** Run Model 1 for $k = 4$ and $\tau = 0.8$ until either $I(t)$ or $S(t)$ drops below $10^{-5}$. Plot $I(t)$, $S(t)$, and $R(t)$ on a single graph. At the end of the computation, report the proportion of the population that became infected and the maximum difference between $I(t) + S(t) + R(t)$ and 1.

Instead of using the equation $dR/dt = I/k$, we could have used the conservation principle

$$I(t) + S(t) + R(t) = 1 \qquad (2)$$

1521-9615/04/$20.00 © 2004 IEEE

for all time. Substituting this for the $dR/dt$ equation gives us an equivalent system of *differential algebraic equations* (DAEs) that we call Model 2.

**Problem 2.** Redo Problem 1 using Model 2. To do this, differentiate the conservation principle and express the three equations of the model as $My' = f(t, y)$, where $M$ is a $3 \times 3$ matrix.

The model has many limitations, but one of them is that the recovery rate is proportional to the current number of infections. This means that we aren't very faithful to the hypothesis that each individual is infected (and infectious) for $k$ days. One way to model this more closely is to use a *delay differential equation* (DDE). We modify Model 1 by specifying that the recovery rate at time $t$ is equal to the rate of new infections at time $t - k$. This gives us Model 3:

$$\frac{dI(t)}{dt} = \tau I(t)S(t) - \tau I(t-k)S(t-k),$$

$$\frac{dS(t)}{dt} = -\tau I(t)S(t),$$

$$\frac{dR(t)}{dt} = \tau I(t-k)S(t-k). \tag{3}$$

One disadvantage of Model 3 is that we must specify initial conditions not just at $t = 0$, but also for $-k \le t \le 0$; thus we need a lot more information. A second disadvantage is that functions $I$, $S$, and $R$ probably will have discontinuous derivatives (for example, at $t = 0$ and $t = k$, when we switch between dependence on the initial conditions and dependence only on the integration history). This causes solvers to do extra work at these points of discontinuity.

**Problem 3.** Redo Problem 1 using Model 3 instead. For $t < 0$, use the initial conditions

$$I(t) = 0, \quad S(t) = 1, \quad R(t) = 0,$$

and let $I(0) = 0.005$, $S(0) = 1 - I(0)$, and $R(0) = 0$. Note that these conditions match our previous ones, but jump at $t = 0$. Compare the three models' results.

### Models that Include Spatial Variation

Epidemics vary in space as well as time. They usually start in a single location and then spread, based on the infected individuals' interactions with their neighbors. Models 1, 2, and 3 lose this characteristic, so now we let $S$, $I$, and $R$ depend on a spatial coordinate $(x, y)$ as well as $t$ and see what such a model predicts.

Because people move in space, we introduce a *diffusion term* that lets infected individuals affect susceptible individuals close to them in space. Diffusion adds a term $\delta((d^2I)/(dx^2) + (d^2I)/(dy^2))S$ to $dI/dt$ and subtracts the same term from $dS/dt$. This produces differential equations analogous to Model 1:

$$\frac{\partial I(t,x,y)}{\partial t} = \tau I(t,x,y)S(t,x,y) - I(t,x,y)/k$$

$$+\delta\left(\frac{\partial^2 I(t,x,y)}{\partial x^2} + \frac{\partial^2 I(t,x,y)}{\partial y^2}\right)S(t,x,y),$$

$$\frac{\partial S(t,x,y)}{\partial t} = -\tau I(t,x,y)S(t,x,y)$$

$$-\delta\left(\frac{\partial^2 I(t,x,y)}{\partial x^2} + \frac{\partial^2 I(t,x,y)}{\partial y^2}\right)S(t,x,y),$$

$$\frac{\partial R(t,x,y)}{\partial t} = I(t,x,y)/k.$$

We assume that the initial values $I(0, x, y)$ and $S(0, x, y)$ are given, that we study the problem for $0 \le x \le 1$, $0 \le y \le 1$, and $t \ge 0$, and that there is no diffusion across the boundaries $x = 0$, $x = 1$, $y = 0$, and $y = 1$.

To solve this problem, we *discretize* and approximate the solution at the points of an $n \times n$ grid. Let $h = 1/(n - 1)$, let $x_i = ih$, $i = 0, \ldots, n - 1$, and let $y_j = jh$, $j = 0, \ldots, n - 1$. Our variables will be our approximations $I(t)_{ij} \approx I(t, x_i, y_j)$ and similarly for $S(t)_{ij}$ and $R(t)_{ij}$.

**Problem 4.**

a. Use Taylor series expansions to show that we can approximate

*(Continued)*

$$\frac{d^2 I(t,x_i,y_j)}{dx^2} = \frac{I(t)_{i-1,j} - 2I(t)_{ij} + I(t)_{i+1,j}}{h^2} + O(h^2).$$

We can derive a similar expression for $d^2I(t, x_i, y_j)/dy^2$.

b. Form a vector $\hat{I}(t)$ from the approximate values of $I(t)$ by ordering the unknowns as $I_{00}, I_{01}, \ldots, I_{0,n-1}, I_{10}, I_{11}, \ldots, I_{1,n-1}, \ldots, I_{n-1,0}, I_{n-1,1}, \ldots, I_{n-1,n-1}$. In the same way, form the vectors $\hat{S}(t)$ and $\hat{R}(t)$, and then derive the matrix $\boldsymbol{A}$ so that our discretized equations become Model 4:

$$\frac{\partial \hat{I}(t)}{\partial t} = \tau \hat{I}(t).*S(t) - I(t)/k + \delta(A\hat{I}(t)).*\hat{S}(t),$$

$$\frac{\partial \hat{S}(t}{\partial t} = -\tau \hat{I}(t).*\hat{S}(t) - \delta(A\hat{I}(t)).*\hat{S}(t),$$

$$\frac{\partial \hat{R}}{\partial t} = \hat{I}(t)/k \qquad (4)$$

where the notation $\hat{I}(t).*\hat{S}(t)$ means that we form the vector from the product of each component of $\hat{I}(t)$ with the corresponding component of $\hat{S}(t)$. To form the approximation near the boundary, assume that the (Neumann) boundary conditions imply $I(t, -h, y) = I(t, h, y)$, $I(t, 1 + h, y) = I(t, 1 - h, y)$ for $0 \le y \le 1$, and similarly for $S$ and $R$. Make the same type of assumption at the two other boundaries.

We can use this model in two ways. First, suppose we fix the time step $\Delta t$ and use Euler's method to approximate the solution. This means we approximate the solution at $t + \Delta t$ by the solution at $t$, plus $\Delta t$ times the derivative at $t$, which gives us an iteration:

$$\hat{I}(t+1) = \hat{I}(t) + \Delta t(\tau \hat{I}(t).*\hat{S}(t) - \hat{I}(t)/k + \delta(A\hat{I}(t)).*\hat{S}(t)),$$

$$\hat{S}(t+1) = \hat{S}(t) + \Delta t(-\tau \hat{I}(t).*\hat{S}(t) - \delta(A\hat{I}(t)).*\hat{S}(t)),$$

$$\hat{R}(t+1) = \hat{R}(t) + \Delta t(\hat{I}(t)/k).$$

This model is very much in the spirit of the models we considered in the last issue—except that it's deterministic instead of stochastic.

Alternatively, we could apply a more accurate ODE solver to this model, as we do in the next problem.

**Problem 5.**

a. Set $n = 11$ (so that $h = 0.1$), $k = 4$, $\tau = 0.8$, and $\delta = 0.2$ and use an ODE solver to solve Model 4. For initial conditions, set $S(0, x, y) = 1$ and $I(0, x, y) = R(0, x, y) = 0$ at each point $(x, y)$, except that $S(0, 0.5, 0.5) = I(0, 0.5, 0.5) = 0.5$. (For simplicity, you need only use $I$ and $S$ in the model; you may derive $R(t)$ from these quantities.) Stop the simulation when the average value of either $\hat{I}(t)$ or $\hat{S}(t)$ drops below $10^{-5}$. Form a plot similar to that of Problem 1 by plotting the average value of $I(t)$, $S(t)$, and $R(t)$ versus time. Compare the results.

b. Let's vaccinate the susceptible population at a rate of $vS(t, x, y)I(t, x, y)/(I(t, x, y) + S(t, x, y))$. This rate is the derivative of the vaccinated population $V(t, x, y)$ with respect to time; we subtract this term from $\partial S(t, x, y)/\partial t$. So now we model four segments of the population: susceptible $S(t)$, infected $I(t)$, recovered $R(t)$, and vaccinated $V(t)$. Your program can track three of these and derive the fourth from the conservation principle $S(t) + I(t) + R(t) + V(t) = 1$. Run this model with $v = 0.7$, and compare the results with those of Model 4.

If you want to experiment further with Model 4, incorporate the delay recovery term in place of $-\hat{I}(t)/k$.

In the models we used in the last issue, we incorporated some randomness to account for any factors not explicitly modeled. We also could put randomness into our differential equation models, resulting in *stochastic differential equations*. (See the "Tools") sidebar for references on this subject.)

### Acknowledgments

I'm grateful to David Gilsinn for explaining delay differential equation models to me.

### Tools

The Matlab function `ode23s` provides a good solver for Problem 1's ordinary differential equations (ODEs). Most ODE software provides a mechanism for stopping the integration when some quantity goes to zero; in `ode23s`, using the Events property in an option vector accomplishes this. Charles van Loan's book[1] provides a good introduction to the numerical solution of ODEs; more specialized texts cover the reasons for preferring a *stiff* solver like `ode23s` for certain types of ODEs.[2]

For Problem 2, we can use ODE software, including `ode23s`, to solve certain differential algebraic equations (DAEs); in Matlab, using the `Mass` property in the option vector accomplishes this. Model 2 is a very simple DAE; Kathryn Brenan, Steven Campbell, and Linda Petzold's book provides more information on the theory and solution of such problems.[3]

Delay differential equations (DDEs) such as those in Problem 3 arise in many applications, including circuit analysis. To learn more, consult a text such as Richard Bellman and Kenneth Cooke's book[4] or Jack Hale and Sjoerd Lunel's book.[5] In Matlab (Release 13), we can solve certain DDEs by using `dde23`.

Stochastic differential equations are an active research area. Desmond Higham[6] gives a good introduction to computational aspects and supplies references for further investigation.

Model 1 is Kermack and McKendrick's SIR model, first introduced in 1927. Nicholas Britton discusses it in more detail.[7]

James Callahan presents the differential equations leading to Model 4,[8] by following a model with one space dimension given in an older text.[9]

### References

1. C.F. van Loan, *Introduction to Scientific Computing*, Prentice Hall, 2000.
2. U.M. Ascher and L.R. Petzold, *Computer Methods for Ordinary Differential Equations and Differential-Algebraic Equations*, SIAM Press, 1998.
3. K.E. Brenan, S.L. Campbell, and L.R. Petzold, *Numerical Solution of Initial-Value Problems in Differential-Algebraic Equations, Classics in Applied Mathematics 14*, SIAM Press, 1995.
4. R. Bellman and K.L. Cooke, *Differential-Difference Equations*, Academic Press, 1963.
5. J.K. Hale and S.M. Verduyn Lunel, *Introduction to Functional Differential Equations*, Springer-Verlag, 1993.
6. D.J. Higham, "An Algorithmic Introduction to Numerical Simulation of Stochastic Differential Equations," *SIAM Rev.*, vol. 43, 2001, pp. 525–546.
7. N.F. Britton, *Essential Mathematical Biology*, Springer, 2003.
8. J. Callahan, *The Spread of a Contagious Illness*, Smith College; www.math.smith.edu/~callahan/ili/pde.html.
9. D.S. Jones and B.D. Sleeman, *Differential Equations and Mathematical Biology*, George Allen and Unwin, 1983.

### Partial Solution to Last Issue's Homework Assignment

### Models of Infection: Person to Person

*By Dianne P. O'Leary*

We have $mn$ patients in a hospital ward, and one of them becomes infected. We track $I(t)$, the proportion of the infected population; $S(t)$, the proportion of the population that never has been infected, and $R(t)$, the remaining proportion. We let $\tau$ be the probability of being infected by a sick neighbor.

**Problem 1.** Run the model for $m = n = 10$, $k = 4$, and $\tau = 0.2$ until there are no infected patients. Plot $I(t)$, $S(t)$, and $R(t)$ in a single graph. If possible, display the epidemic as a movie, where each pixel's color represents a patient's state.

**Problem 2.** Next, we add a probability $\delta$ of patients being moved to a different bed. Modify your model to include mobility and run it for $\delta = 0.01$ until no infected patients remain. Display the results as in Problem 1.

**Problem 3.** Suppose that each day, each susceptible individual has a probability $\nu$ of being vaccinated. Rerun your model with $\nu = 0.1$ until no infected patients remain. Display the results as in Problem 1, and then compare the three models' results.

**Answer:** Figure 1 shows the simulation results for each of these three models. (The Matlab program that generated the results is at www.computer.org/cise/homework.) Generally, mobility increases the infection rate and vaccination dramatically decreases it. In our sample runs, the infection peaks

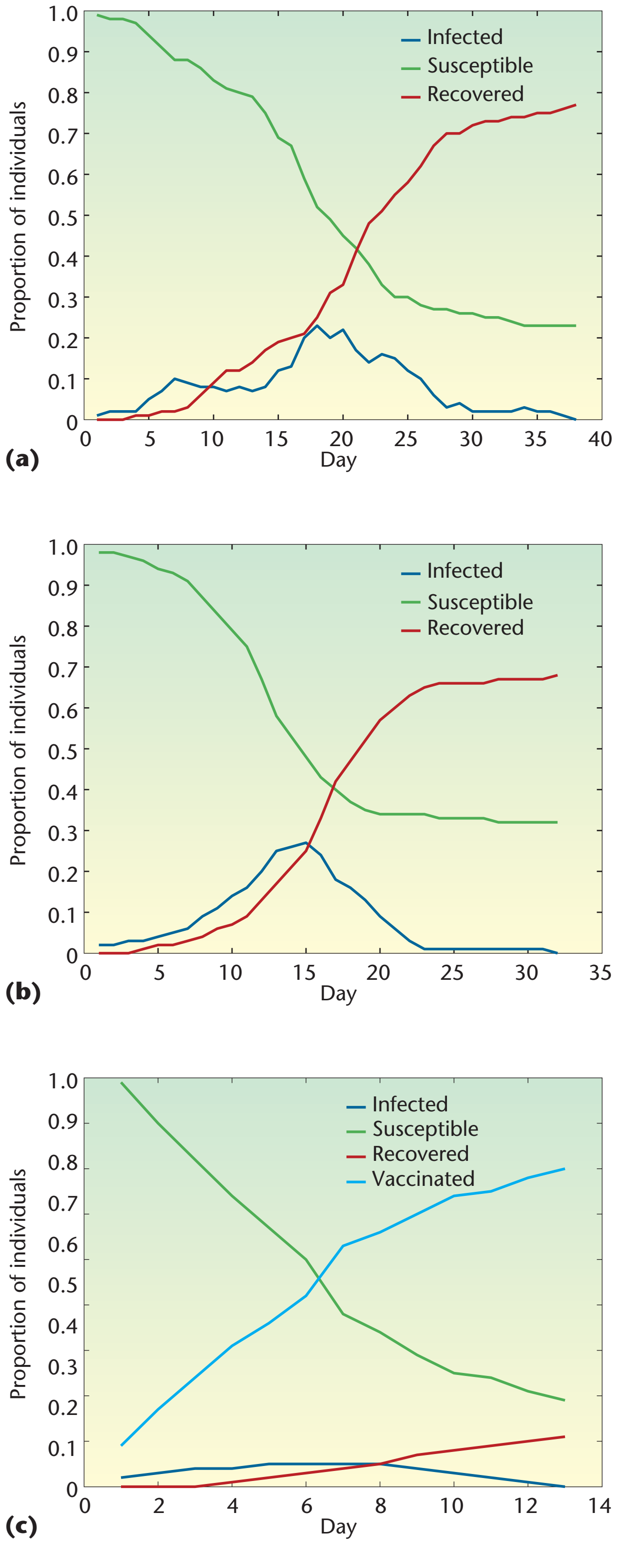

**Figure 1. Proportion of individuals infected by day in a 10 × 10 grid of hospital beds. (a) The infection rate $\tau = 0.2$, (b) the infection rate $\tau = 0.2$ and mobility rate $\delta = 0.01$, and (c) the infection rate $\tau = 0.2$, mobility rate $\delta = 0.01$, and vaccination rate $\nu = 0.1$.**

around day 18 with no mobility, and around day 15 when patients are moved. Individual runs might vary, though.

**Problem 4.** Run Problem 3's model 1,000 times, recording the number of individuals who become infected in each run. (This is equal to the number of recovered individuals when the run is terminated.) Plot this data as a histogram, and then compute the mean number of recovered individuals and the variance in this number. Try several different values of $\nu$ to see whether the variance changes.

**Answer:** Figure 2 shows the histograms for $\nu = 0, 0.1, 0.2$, and 0.3. The mean percent of the population infected drops from 73.6 percent for $\nu = 0$ (with a variance of 4.5 percent) to 4.1 percent for $\nu = 0.3$ (with a variance of only 0.06 percent).

**Problem 5.** Develop a vaccination strategy that will, on average, limit the epidemic to 20 percent of the population. Do this by using a nonlinear equation solver to solve the problem $R(\nu) - 0.2 = 0$, where $R(\nu)$ is the mean number of recovered individuals when we use a vaccination rate of $\nu$. For each value of $\nu$ the solver presents, you will need to get a reliable estimate of $R$ by running the model multiple times. Use Problem 4's variance estimates to determine how many runs to use, and then justify your choice.

**Answer:** From Problem 4, we know that a very low vaccination rate (somewhat less than nu = 0.1) is sufficient to dramatically reduce the infection rate. But using a nonlinear equation solver on a noisy function is quite dangerous; it is easily fooled by outliers, and changing the starting guess, you can make it produce almost any value.

**Problem 6.**

a. Construct the *transition matrix* $\boldsymbol{A}$ corresponding to this Markov chain: element $a_{i,j}$ is the probability of transitioning to state $i$ from state $j$.

b. Let $\mathbf{e}_1$ be the column vector with 1 in position 1 and zeroes elsewhere. If we begin in day one in the first state, then vector $\boldsymbol{A}\mathbf{e}_1$ tells us the probabilities of being in each of the states on day two. Prove this.

c. Similarly, $\boldsymbol{A}^2\mathbf{e}_1$ gives the probabilities for day

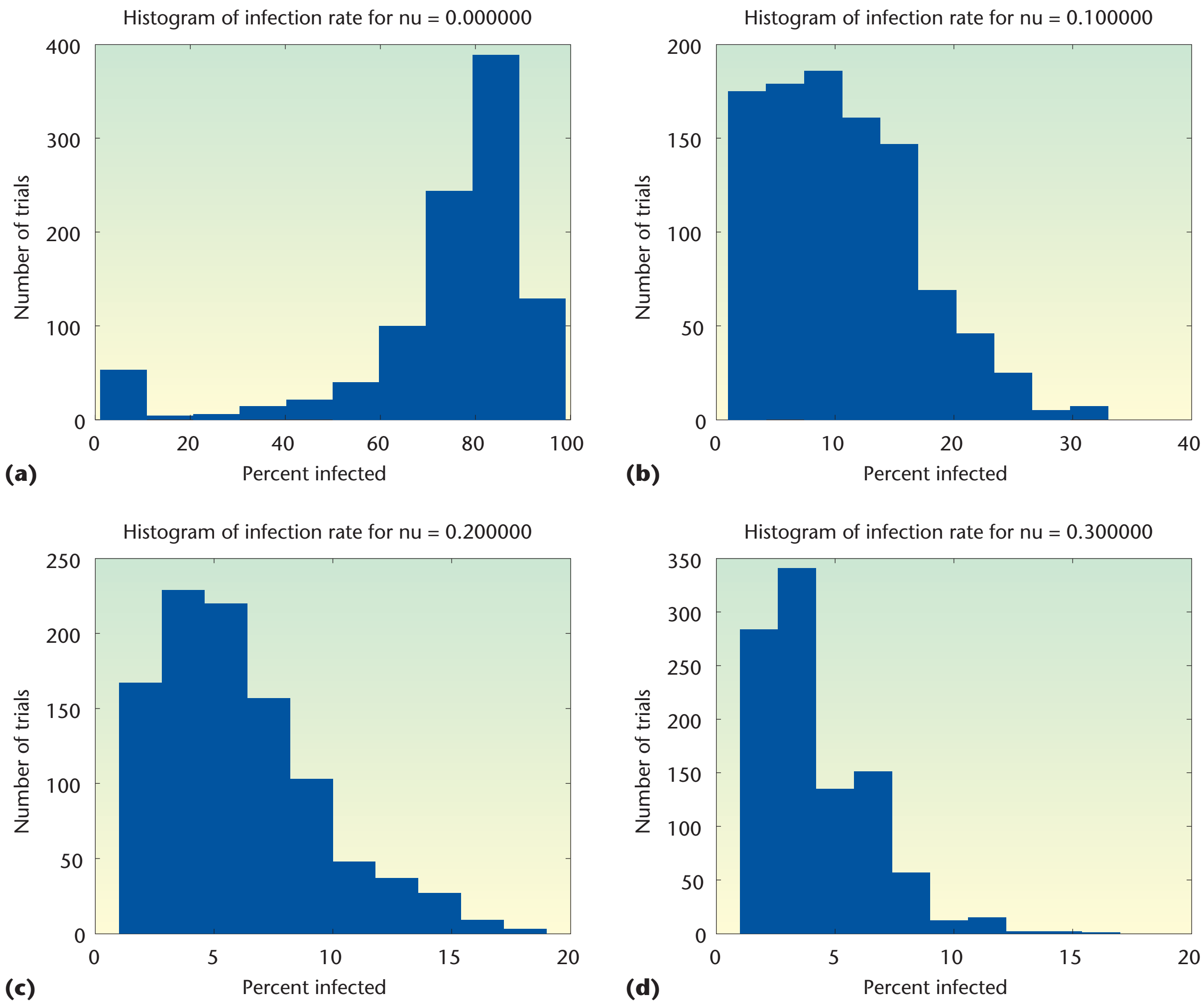

**Figure 2. Results of 1,000 trials for a 10 × 10 grid of hospital beds. The infection rate is $\tau = 0.2$, and the vaccination rate varies.**

three. For efficiency, this should be computed as $A(A\mathbf{e}_1)$ rather than as $(A^2)\mathbf{e}_1$. Explain why, by doing the operations counts.

d. If we compute $\mathbf{z} = A^j\mathbf{e}_1$ for a large enough $j$, we will have the (exact) probabilities of being in each state after the epidemic passes. Use this fact to compute the probabilities of having one, two, or three infected individuals, and compare these probabilities with the results of a Monte Carlo experiment as performed in Problem 4 but using three individuals. How many Monte Carlo simulations does it take to get two digits of accuracy in the probabilities?

e. In this simple problem, you can determine the three probabilities directly from Figure 3, by determining the probability of a transition from state $A$ to states $P$, $Q$, $R$, and $S$. Show how to derive these probabilities, giving the same answer as you obtained via the Markov chain computation earlier.

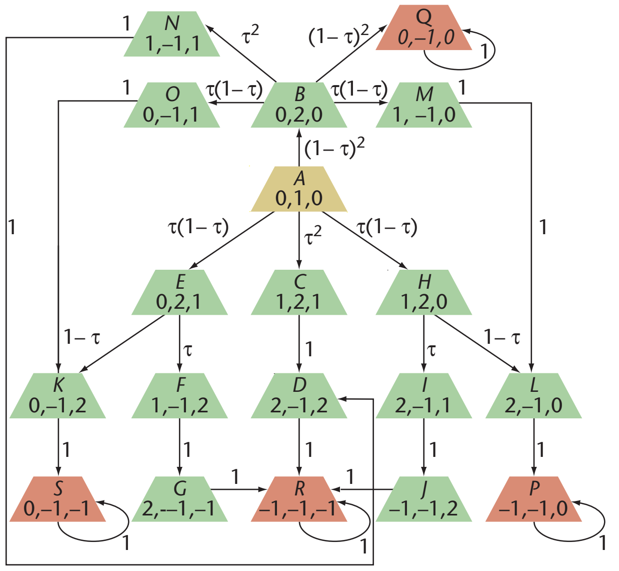

**Figure 3. A Markov chain that models three patients, with the middle patient infected. The yellow state is our starting state; the red states are the possible outcomes when the infection runs its course, corresponding to one, two, or three patients eventually infected.**

**Answer:**

a. Figure 3 gives the transition probabilities; the matrix is given in the Matlab code on the Web site.

b. $\boldsymbol{A}\mathbf{e}_1$ is equal to column 1 of $\boldsymbol{A}$, which contains the probabilities of transitioning from state 1 to any other state. Generally, if $\mathbf{p}$ is a vector of the probabilities of initially being in each of the states, then $\boldsymbol{A}\mathbf{p}$ is the vector of probabilities of being in them at time 1.

c. Computing $\boldsymbol{A}(\boldsymbol{A}\mathbf{e}_1)$ costs $2s^2$ multiplications, where $s$ is the number of states. Computing $(\boldsymbol{A}^2)\mathbf{e}_1$ costs $s^3 + s^2$ multiplications, which grows quite a bit larger when $s$ is large. We should also take advantage of the zeros in $\boldsymbol{A}$ and avoid multiplying by them. If we do this for our matrix, then $\boldsymbol{A}$ has 21 nonzero elements whereas $\boldsymbol{A}^2$ has 23, so again it takes more multiplications to form $(\boldsymbol{A}^2)\mathbf{e}_1$ than to form $\boldsymbol{A}(\boldsymbol{A}\mathbf{e}_1)$. We also should note that the product $\boldsymbol{A}\mathbf{e}_1$ is just the first column of $\boldsymbol{A}$, so we could compute it without multiplications.

d. In this experiment, it took 280 Monte Carlo simulations to get two digits of accuracy. Asking for three digits raises the number of trials into the 10,000s because the variance is high relative to threshold.

e. There is only one path to state $Q$ (corresponding to a single infection), and the product of the probabilities of transitions along this path are $(1 - \tau)^4$. There are two paths to state $S$; summing the product of the probabilities along the paths gives $(\tau(1 - \tau)^2 + \tau(1 - \tau)^3)$. The probability of reaching state $P$ is the same, so the probability of two infections is twice this number. Similarly, the probability of reaching state $R$, corresponding to three infections, is $\tau^2 + 2\tau^2(1 - \tau) + (1 - \tau)^2\tau^2$.

**Dianne P. O'Leary** is a professor of computer science and a faculty member in the Institute for Advanced Computer Studies and the Applied Mathematics Program at the University of Maryland. Her interests include numerical linear algebra, optimization, and scientific computing. She received a BS in mathematics from Purdue University and a PhD in computer science from Stanford. She is a member of SIAM, ACM, and AWM. Contact her at the Computer Science Dept., Univ. of Maryland, College Park, MD 20742; oleary@cs.umd.edu; www.cs.umd.edu/users/oleary/.

The IEEE Computer Society publishes over 150 conference proceedings a year.

Not a member? Join online today!

For a preview of the latest papers in your field, visit www.computer.org/proceedings
